# Unambiguous evidence is required to accurately understand the health impacts of nuclear accidents

**DOI:** 10.1093/jrr/rrz069

**Published:** 2019-11-18

**Authors:** Yurie Kobashi, Toyoaki Sawano, Andy Crump, Masahiro Kami, Masaharu Tsubokura

**Affiliations:** 1 Department of Public Health, Fukushima Medical University School of Medicine, Fukushima City, Fukushima 960–1295, Japan; 2 Kitasato University, Shirokane, Minato-Ku, Tokyo 108-8641, Japan; 3 Medical Governance Research Institute, Takanawa, Minato-Ku, Tokyo 108-0074, Japan

To the editor

Interest in the risk of adverse health effects due to radiation exposure at low doses (<100 mGy) and low-dose rates intensified markedly following Japan’s 2011 Fukushima Daiichi nuclear accident. However, a recent paper entitled ’Nationwide increase in complex congenital heart diseases after the Fukushima nuclear accident’ [1], analysing the increase in congenital heart disease (CHD) in Japan, is extremely troubling for a variety of reasons, particularly as there is no evidence linking the article’s main findings to the actual disaster.

Investigating and verifying the health effects of ionizing nuclear radiation accidents is essential to protect public health, as well as to provide information to countries around the world that may suffer similar events. Knowledge and understanding is critical for emergency preparedness. Japan suffered the joint devastation of the 2011 Great East Japan Earthquake and the subsequent tsunami and nuclear accident at Fukushima, in addition to having experienced the devastating effects of two nuclear bombs in the 1940s. Proper, comprehensive scientific study of all these events and their health impacts are helping to generate a body of evidence upon which the world can base future effective and life-saving policies and plans.

Ionizing radiation can cause a variety of health problems, including genetic damage, birth defects and cancer. Yet studies of female survivors of the Hiroshima and Nagasaki atomic explosions—including those exposed to substantial levels of radiation—found they had children with an incidence of abnormalities similar to the Japanese average. CHD is the most common birth defect and usually has no obvious cause. The authors of the paper confirm that radiation had no detrimental physical effect on congenital abnormalities associated with the Chernobyl nuclear accident, which is supported by a thorough review article [2]. The international consensus is exemplified by the World Health Organization [3], ‘given the low radiation doses received by most people exposed to the Chernobyl accident, no effects on fertility, numbers of stillbirths, adverse pregnancy outcomes or delivery complications have been demonstrated nor are there expected to be any’.

Studies conducted after the Chernobyl accident led various international organizations to conclude that the risk of genetic effects of radiation exposure in the human body was lower than previously thought. In 2007, the International Commission on Radiological Protection (ICRP) determined that, based on animal data, 100 mGy is the limit above which malformations may occur in newborns. Consequently, exposure of intra-uterine fetuses to <100 mGy should not cause teratogenicity.

Many studies have been conducted on the health effects, both direct and indirect, local and national, arising from the Fukushima nuclear accident. Yet assessments of the deleterious impact on both the physical and mental health of local communities—and the nation—remain woefully inadequate. Our knowledge of the short-, medium- and long-term impact of ionizing radiation on human health remains scant and well below the level of what is required. Consequently, slipshod, inexact research can do more harm than good.

Among Fukushima residents, the highest dose recorded was 25 mSv, with the mean dose being 0.8 mSv. At its 64th session in 2017, the United Nations Scientific Committee on the Effects of Atomic Radiation (UNSCEAR) concluded that these doses were too low to affect fetal development or outcome of pregnancy. Thus, in essence, the vague presentation of findings in the paper by Murase *et al*. [1] lacks scientific rigour and appears to be misleading.

The authors examined a national database of the number of surgeries for CHD among <1-year-olds and found the number increased significantly in 2011, remaining at the elevated level for at least three years. However, the aggregated data was not separated by location, individuals, age, level of radiation exposure or other crucial factors. As complex CHD often requires multiple surgeries, the supposed 14% rise identified does not accurately reflect the incidence of CHD, it simply enumerates the actual surgeries performed. The authors went on to identify a correlation between the number of such operations and live birth prevalence, suggesting that heart problems in neonates and infants requiring surgery was increasing, implying a relationship between the increase of complex CHD, live birth rates and the nuclear accident. Yet the nationwide rise in CHD may have no connection whatsoever with the nuclear accident. Indeed, the authors admit that they found no link between radiation exposure in Fukushima and increased levels of CHD, opining that exacerbated maternal stress was a potential cause of their findings, as was diabetes mellitus, which has been a particular and steadily increasing problem in the Fukushima region and which has been linked to several types of CHD. Moreover, no explanation was proffered as to why the localized Fukushima disaster should manifest as a rise in CHD nationwide.

Nationally, the radiation exposure level in 2011 was around the usual value. Data from the Japan Society of Gynecologists’ Congenital Anomaly Monitoring Survey on the incidence of congenital anomalies found no significant differences between Fukushima and other prefectures. In 2014, Fukushima Medical University reported that the incidence of premature births, low birth-weight infants and congenital malformations in the Fukushima area were similar to national figures [4]. Furthermore, Fukushima Health Management Survey data show that the nuclear accident did not affect pregnancy outcomes but did adversely affect maternal mental health [5]. Increases in the Fukushima adult mortality rate due to heart disease, pneumonia, renal failure and diabetes occurred as a consequence of the stress of the disasters and their aftermath, exacerbated by the destruction of local medical facilities and lack of access to health services. Compared to these effects, any increase in CHD caused by radiation exposure would be minimal at best and almost certainly restricted to areas with elevated radiation levels.

The methodology in the paper also appears to be flawed, further weakening its scientific merit, as it is difficult to accurately predict the incidence of any disease simply from the number of surgeries undertaken. Registration of surgeries in Japan is solely for medical evaluation, it cannot provide an accurate indication of the incidence rate of a disease. With respect to CHD, diagnostic methods have improved markedly, especially fetal echocardiography, which has been covered by health insurance since 2010. This has increased the number of children diagnosed, for whom treatment can be started immediately after birth. This may well have increased the number of operations, as patients with complicated CHD often require multiple surgeries. Treatment methods have also advanced, the spread of catheter treatment, use of advanced devices and improved safety all affecting the number of operations undertaken.

Currently, there is little or no epidemiological data supporting any direct relationship between radiation exposure and severe congenital anomalies in humans. The paper by Murase *et al*. attempts to link Japan’s nationwide low-level increase in CHD during 2011–2014 with the Fukushima disaster but provides no credible evidence to support that proposition. As the global Vaccine Hesitancy movement has demonstrated, poorly conceived and sloppy science and analysis, when published in reputable journals or via social media, can actually endanger public health and wellbeing. Although the adverse health effects of Fukushima radiation are predicted to be comparatively minimal, it will be imperative to undertake further and continuing studies to investigate and monitor the effects of radiation on public health in the areas affected. Careful and sound research, expertly scrutinized, needs to be carried out to provide a robust and trustworthy evidence base upon which future public health strategies, policies and action plans can be safely based.

## CONFLICT OF INTEREST

None declared.
